# A 5nW Quasi-linear CMOS Hot-electron Injector for Self-powered Monitoring of Biomechanical Strain Variations

**DOI:** 10.1109/TBCAS.2016.2523992

**Published:** 2016-05-18

**Authors:** Liang Zhou, Adam C. Abraham, Simon Y. Tang, Shantanu Chakrabartty

**Affiliations:** Department of ComputerScience and Engineering, Washington University in St. Louis, St. Louis, mo, 63130 USA; Department of Orthopedic Surgery, Washington University School of Medicine, St. Louis; Department of Orthopedic Surgery, Washington University School of Medicine, St. Louis; Department of ComputerScience and Engineering, Washington University in St. Louis, St. Louis, mo, 63130 USA

**Keywords:** Self-powered Sensors, Piezo-Floating-gate, Hot-electron Injection, Structural Health Monitoring, Biomechanics, Health and Usage Monitoring

## Abstract

Piezoelectricity-driven hot-electron injectors (p-HEI) are used for self-powered monitoring of mechanical activity in biomechanical implants and structures. Previously reported p-HEI devices operate by harvesting energy from a piezoelectric transducer to generate current and voltage references which are then used for initiating and controlling the process of hot-electron injection. As a result, the minimum energy required to activate the device is limited by the power requirements of the reference circuits. In this paper we present a p-HEI device that operates by directly exploiting the self-limiting capability of an energy transducer when driving the process of hot-electron injection in a pMOS floating-gate transistor. As a result, the p-HEI device can activate itself at input power levels less than 5 nW. Using a prototype fabricated in a 0.5-*μ*m bulk CMOS process we validate the functionality of the proposed injector and show that for a fixed input power, its dynamics is quasi-linear with respect to time. The paper also presents measurement results using a cadaver phantom where the fabricated p-HEI device has been integrated with a piezoelectric transducer and is used for self-powered monitoring of mechanical activity.

## I. Introduction

Piezoelectricity driven hot-electron injectors (p-HEI) have been shown to be attractive for long-term, autonomous and self-powered structural health monitoring (SHM) applications [1], [2] where the use of batteries or remote powering is considered to be impractical. Some biomedical applications where the p-HEI sensors have been successfully demonstrated include in-vivo usage monitoring of orthopedic implants [2], [3] and monitoring of mechanical impacts in helmeted sports [4]. Another potential application for self-powered p-HEI sensors, which is the focus of this paper, is for monitoring the activity of load-bearing and biomechanical structures (the musculoskeletal system) in ambulatory animals. The activity statistics recorded by the sensor could potentially be used for understanding the degenerative pathologies of these load-bearing tissues, such as osteoporosis and muscular dystrophy [5], [6], [7], [8], and allow the longitudinal assessment of the efficacy of therapeutics and clinical treatment strategies.

**Fig. 1:**
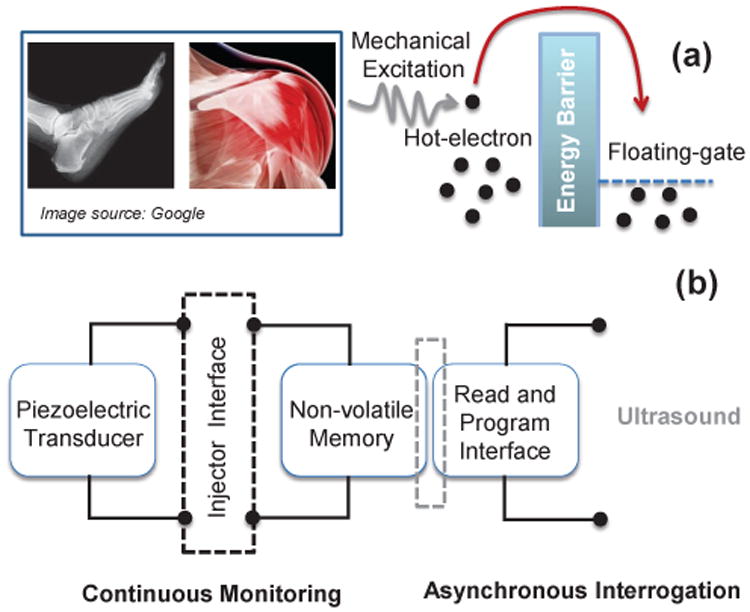
(a) Principle of operation and applications of p-HEI device; and (b) system level architecture of a sensor incorporating the p-HEI device.

While a detailed explanation and verification of the p-HEI device physics have been presented in [9], we briefly introduce the generic working principle of a p-HEI device in Fig. 1(a) using a simplified energy-band diagram. A piezoelectric transducer harvests energy from mechanical strain variations to generate high-energy electrons (or hot-electrons) in the channel of a MOSFET transistor [9]. When the energy of some of these electrons (with the right momentum vector) exceeds the energy barrier (3.2 eV) of the silicon, silicon-di-oxide interface (as shown in Fig. 1(a)), these electrons surmount the barrier and get trapped onto a floating-gate. Because the floating-gate is electrically isolated by a high quality insulating oxide, the injected electrons remain trapped for a long period of time. As the piezoelectric transducer is periodically excited, more electrons are injected onto the floating-gate and the total amount of charge stored on the floating gate is thus a function of the duration and the magnitude of the mechanical excitation. This approach directly couples the physics of piezoelectric energy harvesting with the physics of hot-electron injection to sense, compute and store mechanical usage statistics and hence can be used to push the fundamental limits of self-powered sensing of mechanical strain. The floating-gate also serves as a non-volatile storage memory from which the mechanical usage data could be retrieved at a later stage for offline analysis. This leads to a “sense-now analyze-later” paradigm which has been the basis of p-HEI based sensor architecture [11] as shown in Fig. 1(b). The differentiator between different p-IHEI sensor designs is the interface circuit that connects the piezoelectric transducer with the floating-gate memory. By modifying the topology of the interface circuit, the response of the sensor can be changed and the sensor can also be designed to record different statistics of the strain signal [9], [10]. Fig. 1(b) also shows the read-out and initialization interface of the p-HEI sensor. Because read-out and initialization of the floating-gates require a more precise calibration and control, which requires more energy than what can be harvested from mechanical strain variations, the functionality is powered by a different energy source, as shown in Fig. 1(b). This source of energy could be delivered either using a plug-and-play interface [4], or using a radio-frequency telemetry link [11] or using an ultrasonic telemetry link [12]. Because the focus of this paper is on the design of the p-HEI interface circuitry we will use a plug-and-play interface for read-out, calibration and programming, keeping in mind that the approach can be integrated with other remote powering techniques.

**Fig. 2:**
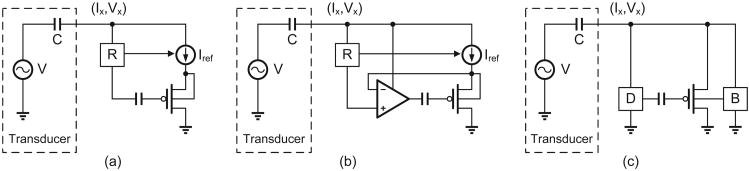
Configuration of p-HEI injectors: (a) constant current injector reported in [9]; (b) linear injector reported in [10]; and (c) proposed injector. The modules R, D and B in the figures refer to bias generation circuits.

In literature, two topologies of p-HEI interface circuitry have been proposed and are shown in Fig. 2(a) and (b). For both these topologies, the energy harvested from the piezoelectric transducer (whose strain-mode equivalent circuit is shown in Fig. 2) is rectified and then used for generating current and voltage references labeled as “R” in Fig. 2(a) and (b). The rate at which electrons are injected on the floating-gate is determined by the transistor source current and by the channel-to-drain voltage [9], [10]. In the topology shown in Fig. 2(a), the source current is limited by a constant current reference which yields an electron injection rate that exponentially decreases with respect to time [9]. The compressive response of the injector with respect to time makes it challenging to discriminate between different mechanical usage statistics accumulated over a relatively short duration. The topology shown in Fig. 2(b) overcomes this problem by using a negative feedback approach to linearize the hot-electron injection process [10]. A feedback amplifier and a current reference maintains the source current and the channel-to-drain voltage constant which ensures a constant injection current. This yields a linear response which has been exploited for designing self-powered strain-gauges [13] and self-powered mechanical impact counters [4]. However, the disadvantage of both these approaches is that most of the transducer energy is dissipated in the rectification and reference generation circuitry. In [9] it was shown that the p-HEI process is functional even at picoampere current levels, which implies that a better interface circuitry connecting the piezoelectric transducer to the floating-gate transistor could significantly reduce the power dissipation of the p-HEI sensor. Lowering the power dissipation of the p-HEI sensor will make it attractive for monitoring applications that require ultra-low activation levels of mechanical strain and mechanical acceleration. For example in systems monitoring seismic activity, the frequency range of the oscillations is usually below 1 Hz, and acceleration level is less than 1 *m/s*^2^ [14]. An embedded piezoelectric accelerometer with a tip-mass of 1 *mg* and an oscillation speed 1 *cm/s* could generate electrical power only in the range of a few nanowatts.

Fig. 2(c) illustrates the proposed topology that directly connects the transducer to the floating-gate transistor and exploits the limited driving capability of the energy transducer. The additional biasing modules labeled as “D” and “B” shown in Fig. 2(c) are not in the direct path of energy transfer between the transducer and the floating-gate transistor. As a result, the proposed p-HEI sensor could in principle operate at energy levels that is an order of magnitude lower than previously reported injector topologies. The description of the proposed injector is organized in this paper as follows: section II presents a mathematical and numerical model describing the operation of the proposed p-HEI injector followed by section III where the models are validated using measured results obtained from a fabricated injector prototype. Section IV presents measurement results where the injector is integrated with a piezoelectric cantilever beam and the resulting p-HEI sensor is shown to measure and record mechanical usage statistics. Section V concludes the paper with a discussion on techniques that could extend the functionality of the proposed injector.

## II. Mathematical Model of the p-HEI Device

The mathematical model presented in this section is based on the schematic shown in Fig. 2 where it is assumed that the output of the energy transducer is characterized by its output voltage and output current (*V_x_*(*t*), *I_x_*(*t*)). Since the focus of this paper is to determine the limits of energy-efficiency (low levels of input power), the floating-gate pMOS transistor in Fig. 2(c) will be assumed to be always biased in the weak-inversion regime. Also, for the sake of simplicity we will assume that the drain of the pMOS transistor is connected to ground. The transistor source current *I_s_* = *I_x_* can then be expressed in terms of the floating-gate voltage *V_fg_*, the substrate voltage *V_B_* and the source voltage *V_x_* as [15]

(1) $$I_{x}=I_{0}\exp[\frac{(1-\kappa )V_{B}}{\kappa U_{T}}]\exp(\frac{-V_{fg}}{\kappa U_{T}})[\exp(\frac{V_{x}}{U_{T}})-1]$$

where *I*_0_ is the characteristic current, *κ* is the gate-efficiency factor and $U_{T}=\frac{\kappa T}{q}$ is the thermal voltage (≈26 mV at 300 K) which is directly proportional to the ambient temperature. In weak-inversion regime, the hot-electron injection current can be expressed by the following empirical model [16] as

(2) $$I_{inj}=-\beta I_{x}\exp(V_{x}/V_{inj})$$

where *β* and *V_inj_* are injection parameters which are a function of the transistor size and process parameters. The injection current changes the charge on the floating-gate *Q_fg_* and the floating-gate voltage *V_fg_* according to

(3) $$C_{T}\frac{dV_{fg}}{\text{dt}}=I_{inj}$$

where *C_T_* = *C_cg_*+*C_tun_*+*C_gs_* denotes the total capacitance at the floating-gate node with *C_cg_* being gate-coupling capacitor, *C_tun_* being the tunneling capacitor and *C_gs_* being the gate-to-source capacitance. To complete the model of the injector circuit in Fig. 5, the voltage *V_B_* is chosen such that it tracks the highest of the source or the drain voltages. For the sake of simplicity we will use a smooth logistic function that interpolates *V_B_* between *V_x_* and ground as

(4) $$\frac{V_{B}}{U_{T}}=\log(1+\exp(\frac{V_{x}}{U_{T}})).$$

The equations 1-4 are coupled which makes it difficult to infer a closed-form solution of *V_fg_*(*t*) for a general form of the input *V_x_*(*t*), *I_x_*(*t*). Fortunately, closed-form expressions for *V_fg_*(*t*) can be obtained under special conditions which will provide the justification for the design of the proposed injector. The first special case is when the output current of the transducer *I_x_*(*t*) = *I_x_* is kept constant; and the second case is when the output voltage of the transducer *V_x_*(*t*) = *V_x_* is kept constant.

### A. Constant current mode

**Fig. 3:**
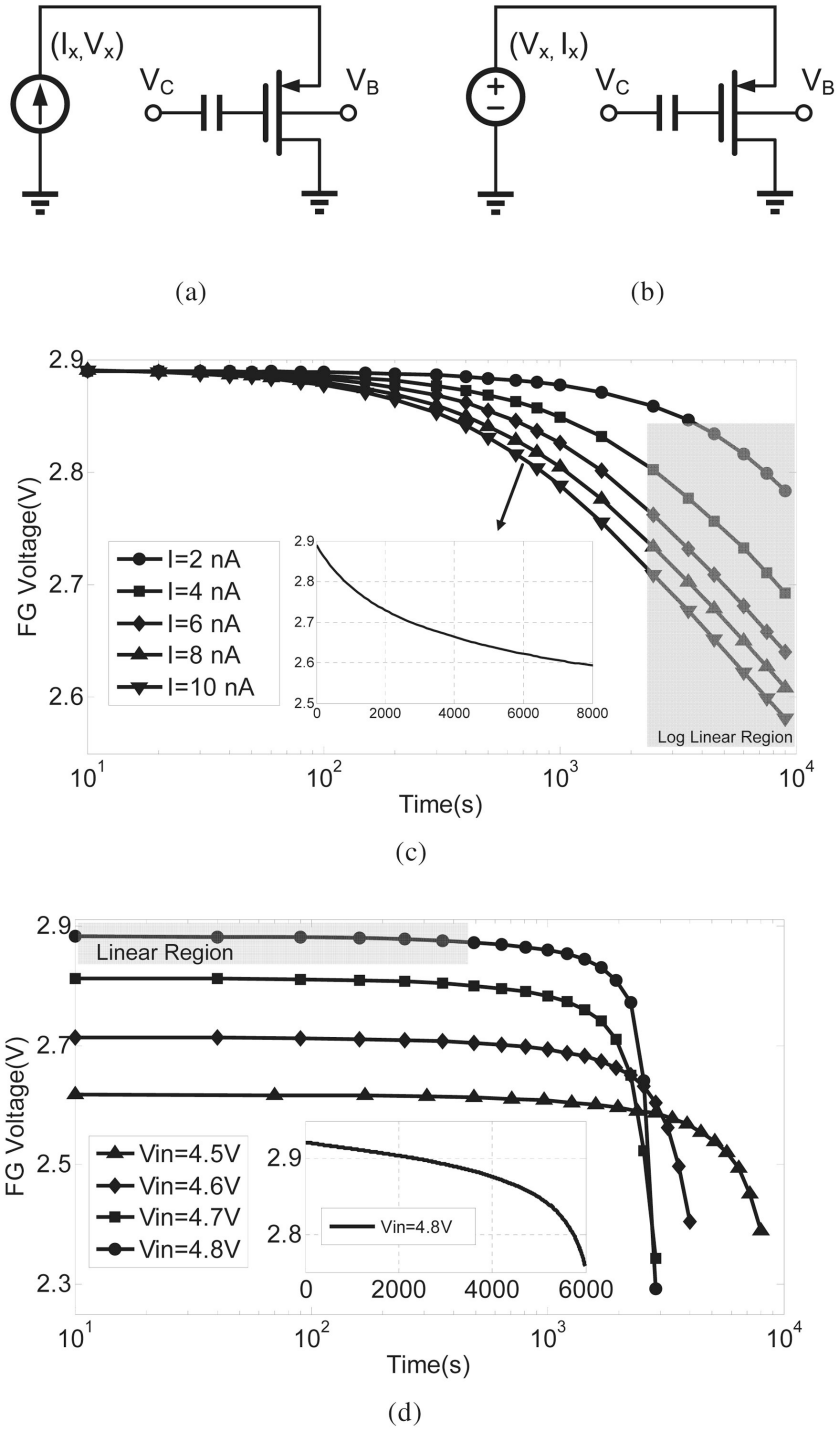
Specific operational modes of the injector:(a) constant current mode; and (b) constant voltage mode. Measured results showing (c) a saturating response corresponding to the constant current mode (a); and (d) exponential response corresponding to the constant voltage mode (b).

When the injector is driven by a constant current *I_x_* as shown in Fig. 3(a) the equations 1-4 reduces to

(5) $$V_{fg}(t)=V_{fg0}-V_{inj}\log(1+K_{1}t)$$

where *V_fg_*_0_ is the initial floating-gate voltage and the values of *K*_1_ is given by

(6) $$K_{1}=\frac{\beta I_{x}}{V_{inj}C_{T}}{\left(\frac{I_{x}}{I_{0}}\right)}^{\frac{\kappa U_{T}}{V_{inj}}}\exp(\frac{V_{fg0}}{V_{inj}})$$

Note that the value of *K*_1_ is positive. Therefore, the injection process, according to equation 5 is a self-stabilizing negative-feedback process. Fig. 3(c) shows the *V_fg_*(*t*) measured from fabricated injector prototypes showing consistency with the equation 5. The inset in Fig. 3(c) shows the response on a linear time-scale clearly showing the self-stabilizing response. Note for large *t*, equation 5 can be approximated by a log-linear form as

(7) $$V_{fg}(t)\approx V_{inj}\log\{\frac{V_{inj}C_{T}}{\beta I_{x}}{\left(\frac{I_{x}}{I_{0}}\right)}^{-\frac{\kappa U_{T}}{V_{inj}}}\}-V_{inj}\log(t).$$

### B. Constant voltage mode

The coupled differential equations 1-4 can also be solved in closed form when the output voltage of the transducer is assumed to be constant *V_x_*(*t*) = *V_x_*, as shown in Fig. 3(b). In this operating mode, the hot-electron process shows a positive feedback response, where decrease in the floating-gate voltage *V_fg_* leads to an increase in the pMOS source current which further increases the hot-electron injection rate. The form of *V_fg_*(*t*) can be obtained from equation 1-4 as

(8) $$V_{fg}(t)=V_{fg0}+\kappa U_{T}\log(1-K_{2}t)$$

where

(9) $$K_{2}=\frac{\beta I_{0}}{\kappa C_{T}U_{T}}\exp(\frac{V_{x}}{\kappa U_{T}}+\frac{V_{x}}{V_{inj}}-\frac{V_{fg0}}{\kappa U_{T}})$$

Fig. 3(d) shows the injector's response when the transducer output voltage is varied from 4.5 V to 4.8 V. At the start, when the injection duration *t* is small, the approximation *ln*(1−*x*) ≈ −*x* can be used in equation 8 to obtain

(10) $$V_{fg}(t)=V_{fg0}-\kappa K_{2}U_{T}t$$

which is linear with respect to *t*. However, as more electrons are injected and the floating-gate voltage *V_fg_* reduces, the injection rate increases to the point where the change in *V_fg_* is exponential which is also illustrated in the inset of Fig. 3(d).

**Fig. 4:**
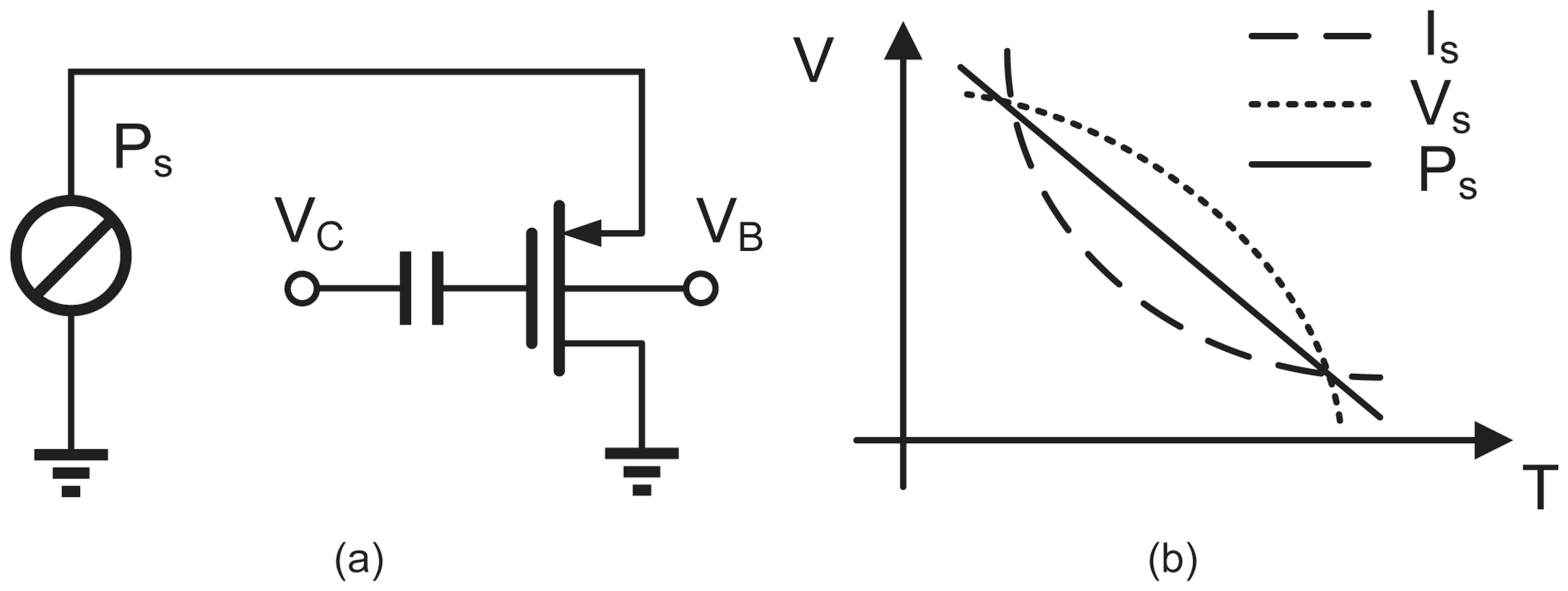
Interpolation of constant-current injection and constant-voltage injection leading to the proposed quasi-linear constant-power injection.

The operation of the proposed hot-electron injector is illustrated in Fig. 4 and is based on the following two observations: (a) constant current mode hot-electron injection is a negative-feedback process and *V_fg_*(*t*) shows a saturating response as shown in Fig. 3(c); and (b) constant voltage mode hot-electron injection is a positive-feedback process where *V_fg_*(*t*) decreases exponentially as shown in Fig. 3(d). Thus, when the input power is constrained to be constant (or product of input voltage and input current is constant) the hot-electron injection should interpolate between both the negative and positive feedback process, as illustrated in Fig. 4, leading to a quasi-linear response.

### C. Constant power injector model and numerical study

However, when the input power to the injector is held constant, equations 1-4 can not be solved in closed form and we resort to numerical analysis using a specific type of energy transducer model. Since the target application for the injector device is for monitoring mechanical activity, a piezoelectric cantilever model has been chosen for this study.

**Fig. 5:**
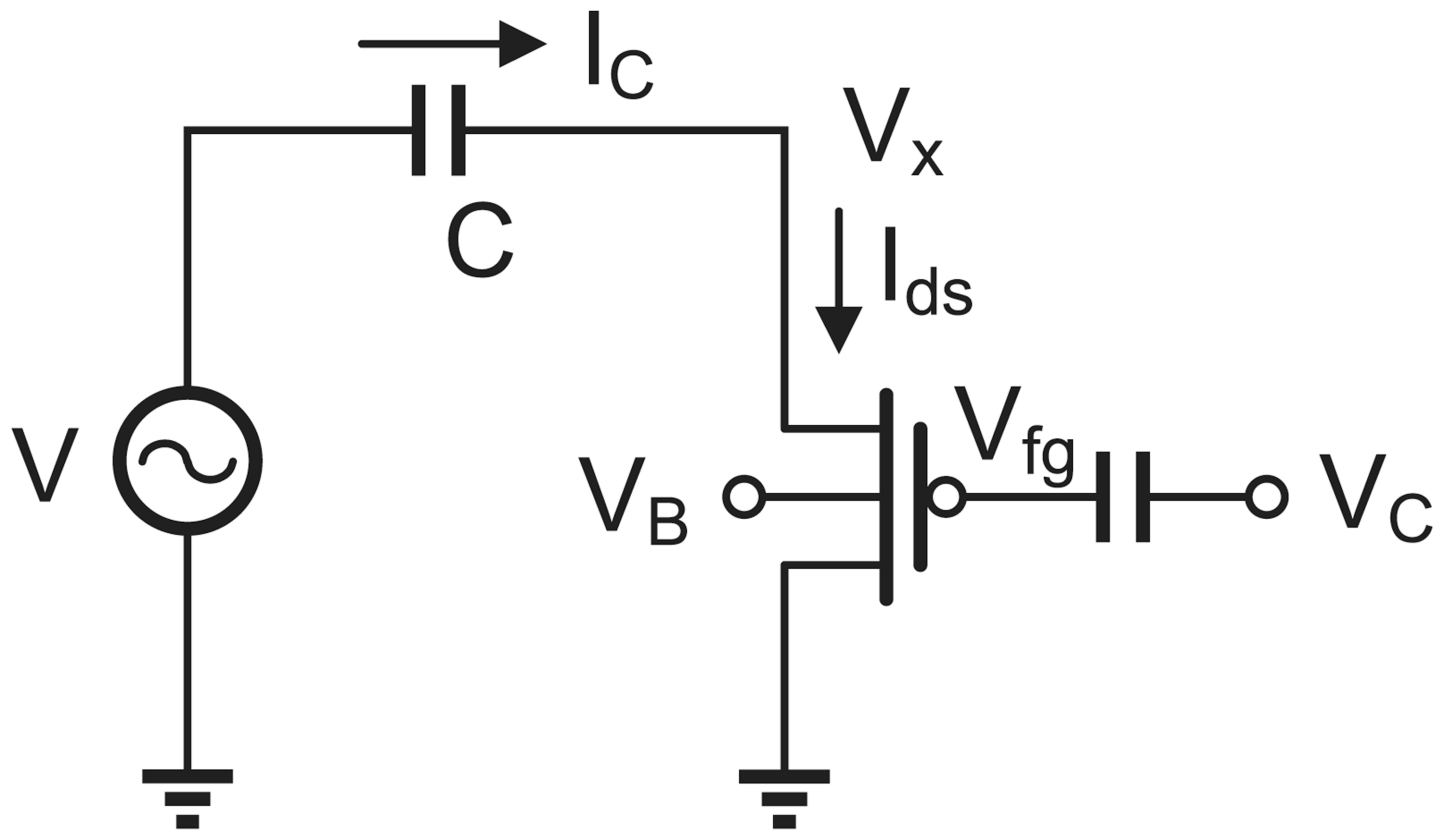
Simplified model of an injector driven by a piezoelectric transducer.

A simplified equivalent circuit model of a piezoelectric transducer operating in strain-mode (non-resonance mode) and driving the process of hot-electron injection in a pMOS transistor is shown in Fig. 5. The voltage source *V* models the electrical signal transduced by the strain variations and the capacitor *C* models the mechanical stiffness of the transducer. Both these variables are a function of the dimensions, the material properties and the mechanical configuration of the piezoelectric transducer. For a cantilever configuration and with a transducer with dimensions *w*×*l*×*h*, the open-source voltage (V) generated can be expressed in terms of the mechanical force (F) perpendicular along the length as [17]:

(11) $$V=\frac{F}{w}g_{31}=S(t)Y^{E}hg_{31}=\frac{S(t)Y^{E}d_{31}h}{\varepsilon_{p}}$$

where *g*_31_ and *d*_31_ are piezoelectric constants, *S*(*t*) is the time-varying mechanical strain, *Y^E^* is the short circuit elastic modulus and *ε_p_* is the electrical permittivity of the material. The capacitance in the equivalent circuit model is given by

(12) $$C=\varepsilon_{p}\frac{w\times l}{h}$$

A change in mechanical strain induces a displacement current through *C* which is expressed as

(13) $$I_{C}=C(\frac{dV}{\text{dt}}-\frac{dV_{X}}{\text{dt}})$$

where *V_X_* is the output voltage of the transducer.

The next step in the numerical analysis is to determine the values of the model parameters in equations 1-4 using experimental data. The measured injector response in either constant current mode or constant voltage mode could be used to estimate the parameters *K*_1_ and *K*_2_ which can then be used to estimate the parameters of the model in 1-4. The estimated model parameters are summarized in Table I which also summarizes the equivalent circuit parameters for the piezoelectric cantilever obtained from [18].

**Fig. 6:**
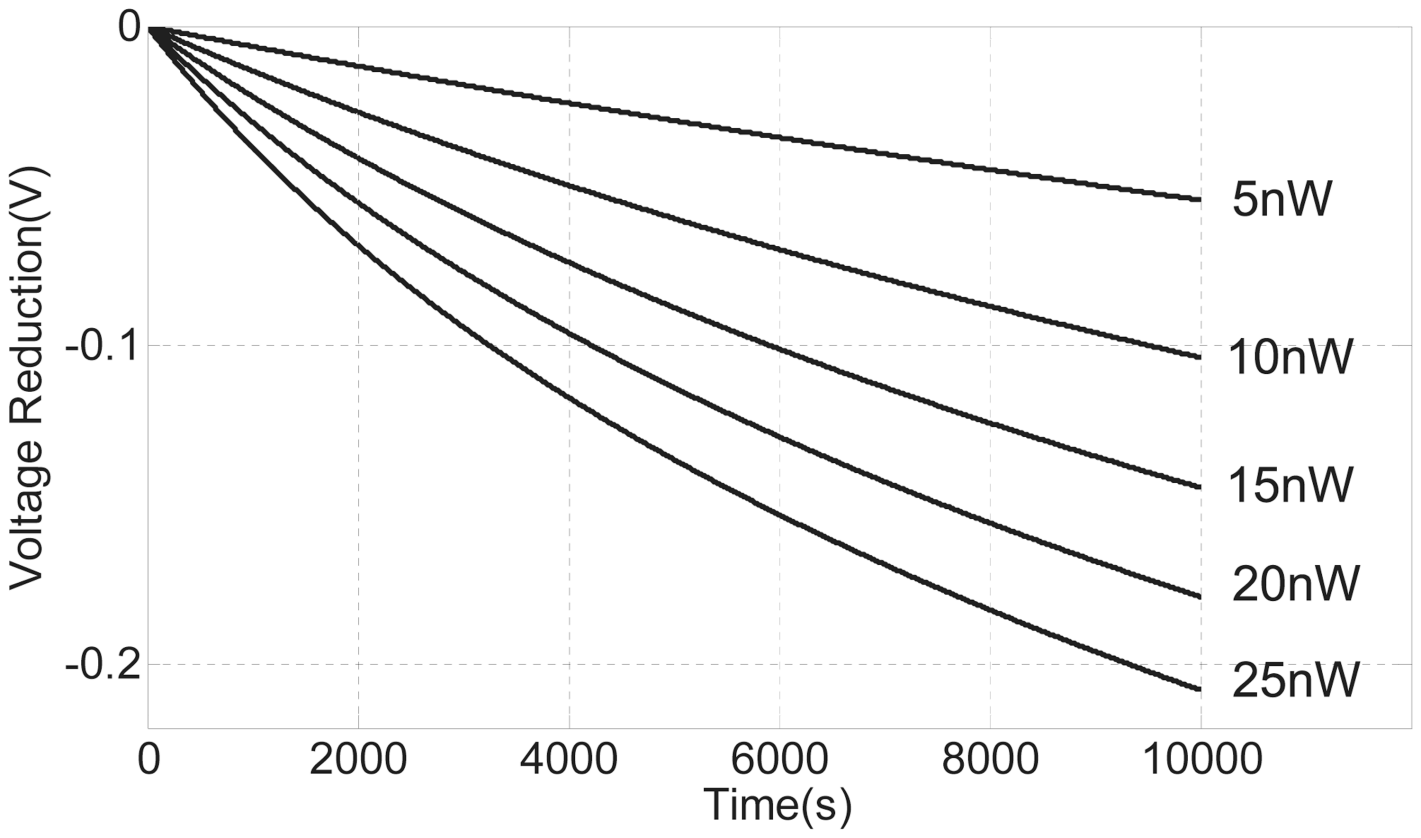
Simulated results showing injector responses at different levels of input power.

**Fig. 7:**
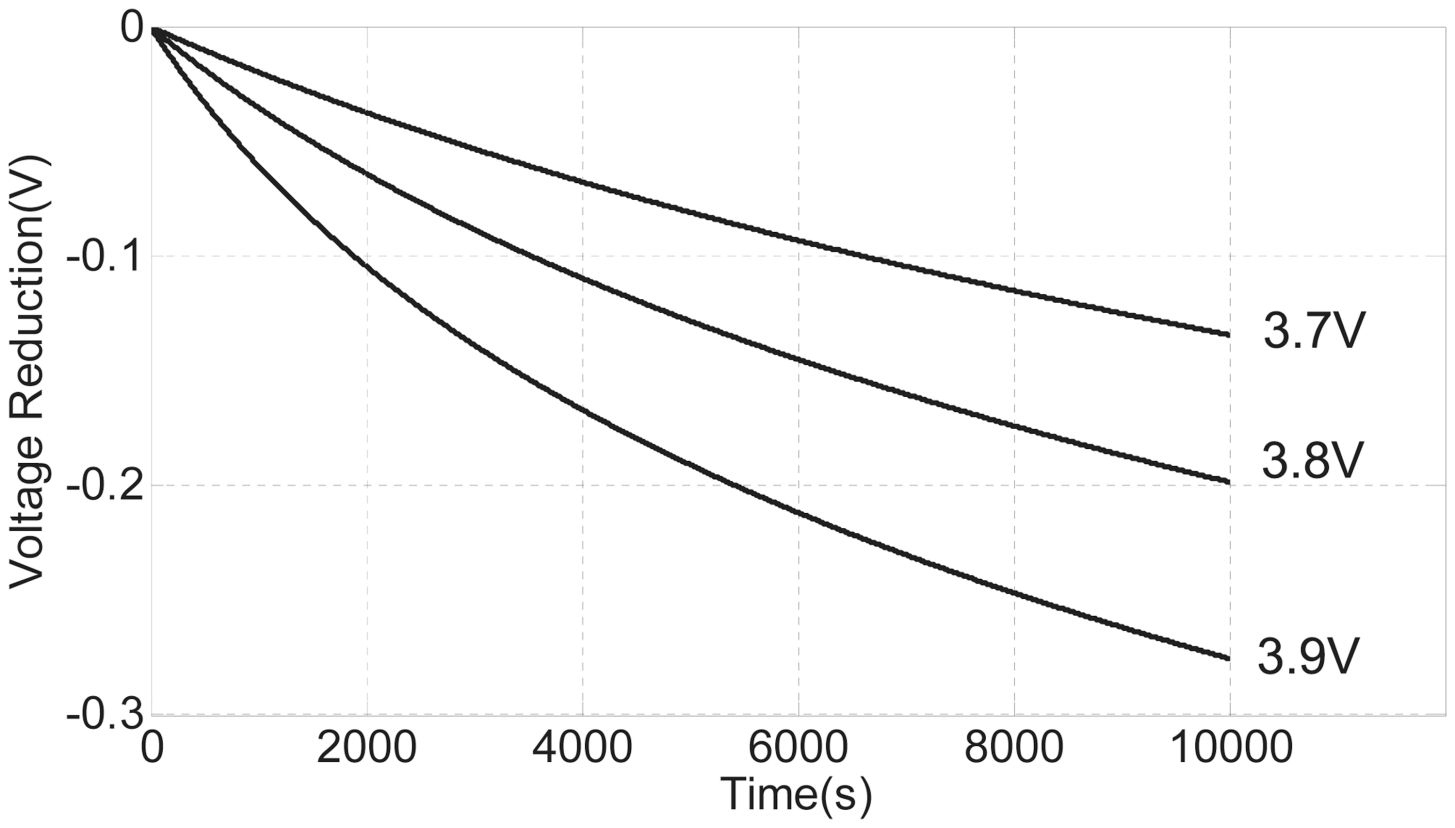
Simulated results showing injector responses different initial conditions on *V_fg_* for constant input power (*P_in_* = 15*nW*).

For the sake of simplicity, *V*(*t*) was chosen to be a ramp function in a manner that the average input power to the injector can be varied. Fig. 6 shows the simulated response of the injector where the reduction in floating-gate voltage is plotted for different levels of input power. As predicted, the response is quasi-linear which is indeed an interpolation of the constant current and constant voltage mode responses. The simulation study also shows that the injector can be operated at power levels in the range of a few nano-watts. In another simulation study shown in Fig. 7, the response of the injector is plotted for different initial values of the floating-gate voltage and at a fixed input power level of 15 nW. The result shows that the duration of the injector can be programmed using different initial values of the floating-gate voltages. This implies that the injector can be used for applications with different requirements on the duration of monitoring.

## III. System Implementation and Measurement Results

### A. Schematic of the Injector

**Fig. 8:**
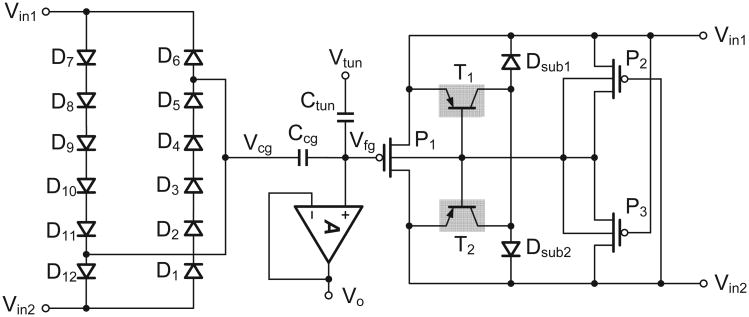
Schematic implementation of the low-power injector.

Fig. 8 shows the complete schematic of the proposed injector implemented on a standard 0.5-*μ*m bulk CMOS process. The transistor pair *P*_2_ and *P*_3_ implements the interpolation function as in equation 4 and ensures that the substrate of *P*_1_ is maintained at the highest potential. The diodes *D*_1_ to *D*_12_ serve two functions: (a) they limit the input voltage *V_x_* to be within a compliant operating range; and (b) they generate the bias for the control-gate voltage $V_{cg}\approx \frac{V_{in}}{m}$, where *m* is the number of the diodes in series. *T*_1_ and *T*_2_ represent the parasitic transistors formed by the source and drain terminals with the bulk and p-substrate. The diodes *D_sub_*_1_ and *D_sub_*_2_ maintain the substrate at the lowest potential.

One of the considerations for implementing the injector in a bulk CMOS process is that the nwell and p-implant of a normal *p-n* diode can form a parasitic *p-n-p* transistor with the p-type substrate. This configuration draws a significant current even when the transducer output voltage *V_in_* is small. This issue can be resolved using a diode-connected pMOS with an isolated and shielded nwell.

A unity-gain buffer is used for reading out the voltage on the floating-gate node as shown in Fig. 8. To prevent the floating-gate charge from leaking to the oxide through the vias, the FG node is formed using the first polysilicon layer and is connected to the input of the amplifier through the same poly layer. To avoid hot-electron injection through the gate of the input transistors of the read-out amplifier, the voltage of the FG is maintained below 3.3 V. Also, when the injector is self-powered through the energy transducer, the power to the readout buffer is disabled.

**Fig. 9:**
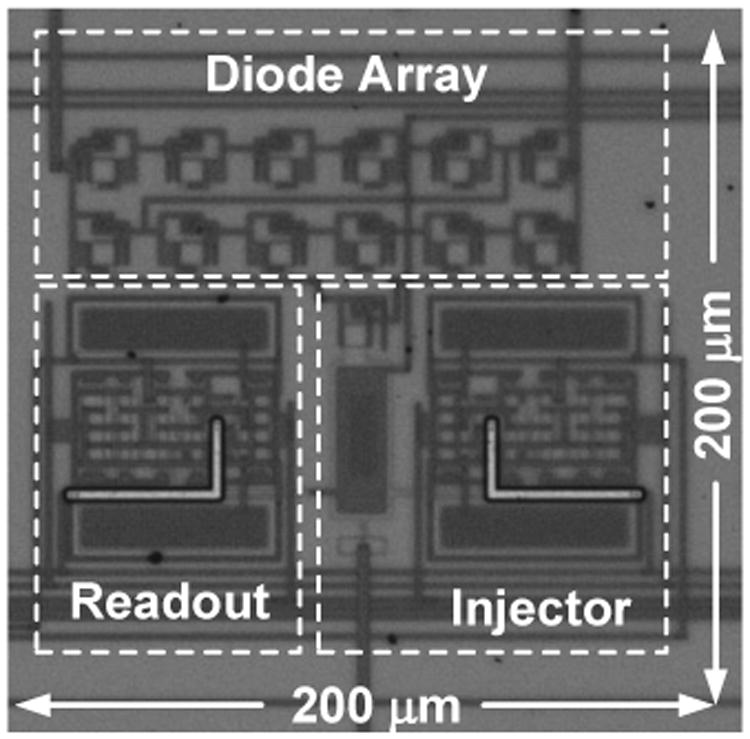
Micro-photograph of the chip die.

The injector was prototyped in a standard 0.5-*μ*m CMOS process using circuit components whose form factors are summarized in the table II. The circuit occupies an area of 200 *μ*m × 200 *μ*m and its die photograph is shown in Fig. 9.

### B. Measurement Results

**Fig. 10:**
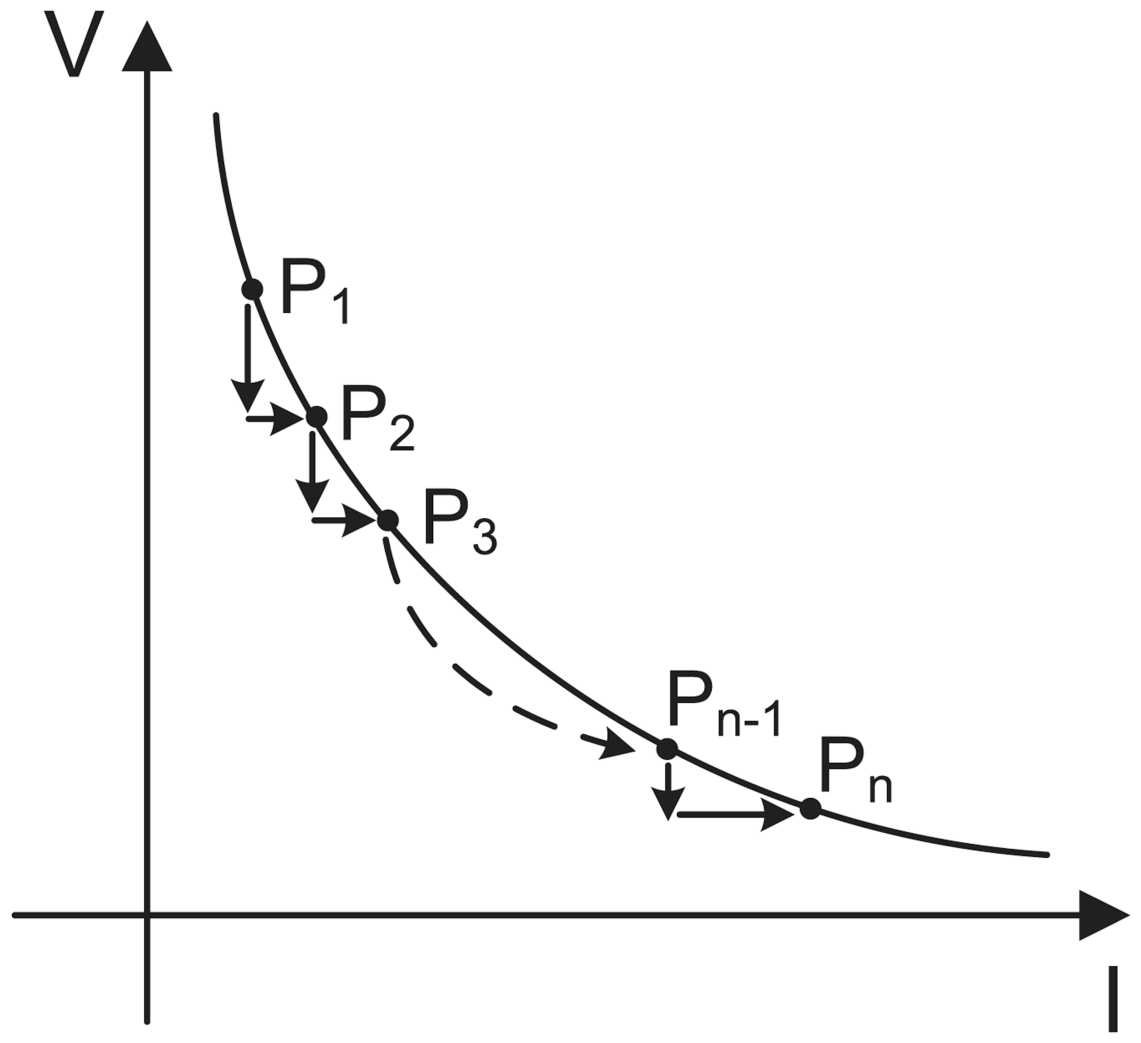
Measurement technique used to maintain a constant input power level using a source-meter

**Fig. 11:**
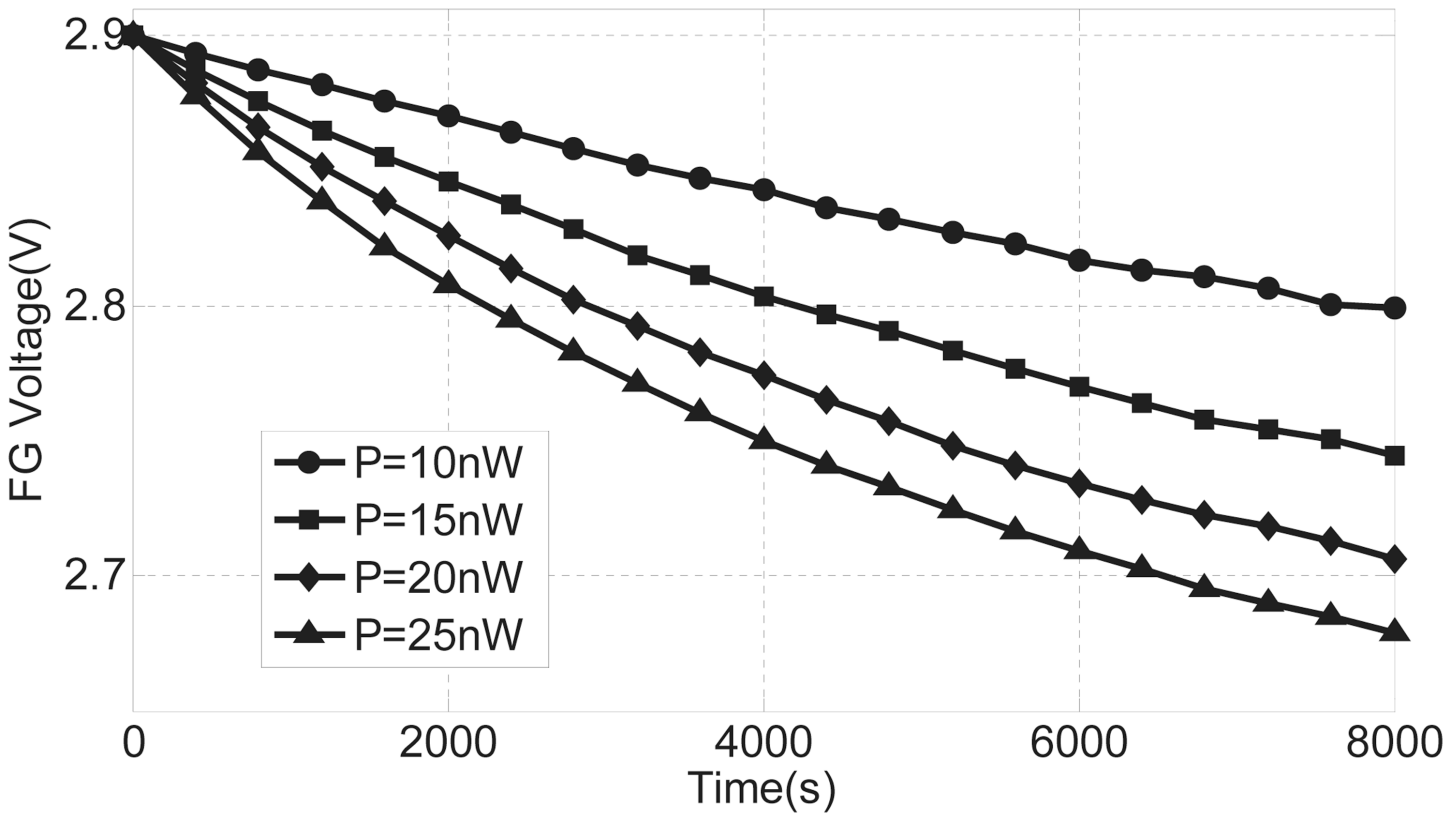
Measured injector responses for different levels of input power.

The fabricated prototypes were used to measure the injector responses for different levels of input power. The floating-gate of the injector was calibrated to a fixed initial voltage. A Keithley 2400 source meter was then used to directly power the injector. A constant input power was maintained by continuously adapting the source current as illustrated using a constant power curve in Fig. 10. As more electrons are injected onto the floating-gate, its voltage reduces leading to a reduction in the source voltage. After each 10 second duration, the reduction in source voltage is measured using the source-meter and the source current is increased so that the resulting input power *P_in_* remains constant. Fig. 11 shows the measured injection responses when the input power is varied from 10 nW to 25 nW. The measured results validate the quasi-linear responses as predicted using simulations shown in Fig. 6.

**Fig. 12:**
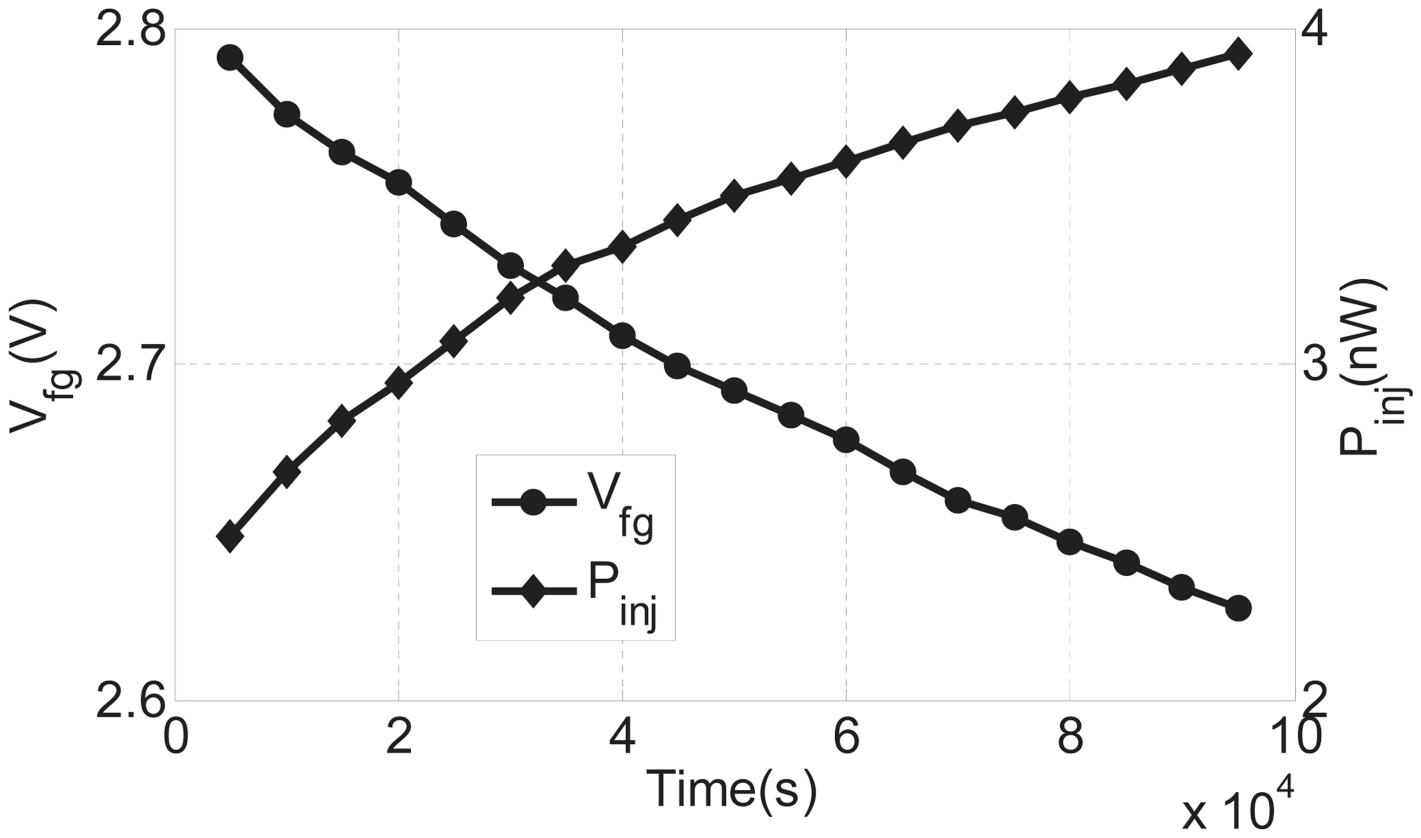
Injector response and power consumption of the injector with fixed input power=5 nW.

Note that for the fabricated injector, the diode chain also consumes a portion of the input power. This can be estimated using the expression of the diode current as

(14) $$I_{dio}=I_{0}\exp(\frac{V_{in}}{m\kappa U_{T}})$$

with *m* being the number of diode in the chain. For this implementation *m* = 6 which implies that for *V_in_* less than 4.5 V, the diode leakage current is smaller than 500 pA. The actual power consumed by the injector was obtained by subtracting the power consumed by the diode chain estimated using the measured results shown in Fig. 12. The response of the injector was obtained at an input power level of *P_in_* = 5*nW* which results in a longer monitoring duration. The measured result again validates the quasi-linear response, which is consistent with the numerical analysis presented in Section II.

## IV. Verification of the Injector

**Fig. 13:**
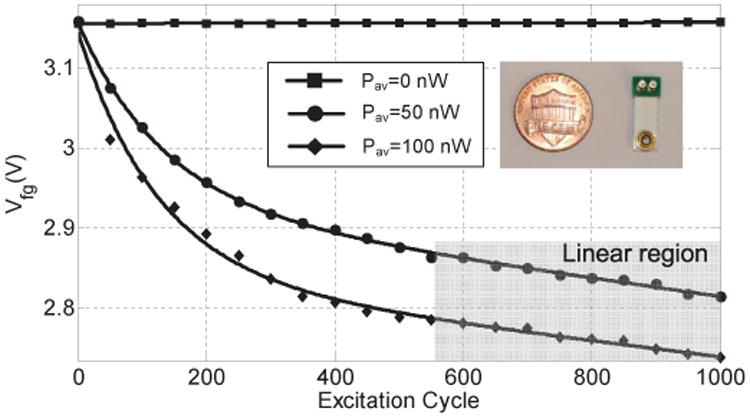
Measured injector responses for different levels of power generated by the piezoelectric cantilever.

**Fig. 14:**
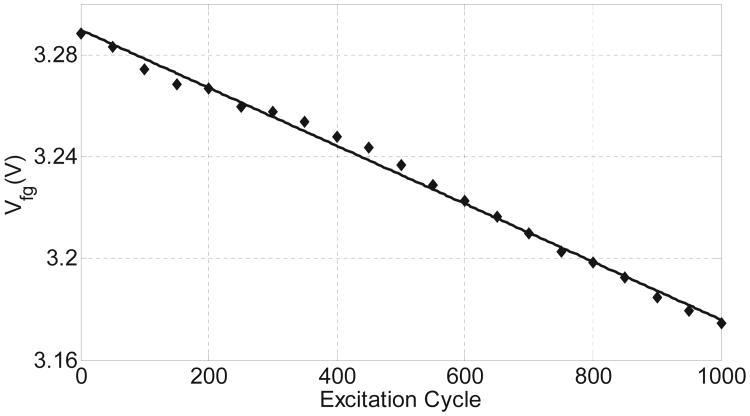
Measured injector response when the power generated by the piezoelectric cantilever is pushed down to 5nW.

The fabricated injector prototype was integrated with a miniature commercial off-the-shelf (COTS) piezoelectric cantilever (dimensions: 10mm×5mm×0.1mm, material:PZT-5H) as shown in Fig. 13 (inset). The cantilever was subject to periodic mechanical excitation (step displacement) such that it generated different levels of output power. To measure the power delivered by the transducer, a resistive load was connected to the output of the cantilever. For a specific mechanical excitation pattern, the voltage was measured across the resistive load which was then used to determine the output power level. The resistor was then replaced by the fabricated injector and transducer was again subjected to similar mechanical excitation. The measured injector responses are shown in Fig. 13 for two levels of output power which again validates the predicted quasi-linear responses. In another set of experiment, the mechanical excitation of the transducer was reduced to a level such that the output was around 5 nW. The measured response for this condition is shown in Fig. 14 and clearly demonstrates a linearized response, as predicted by the simulation model.

**Fig. 15:**
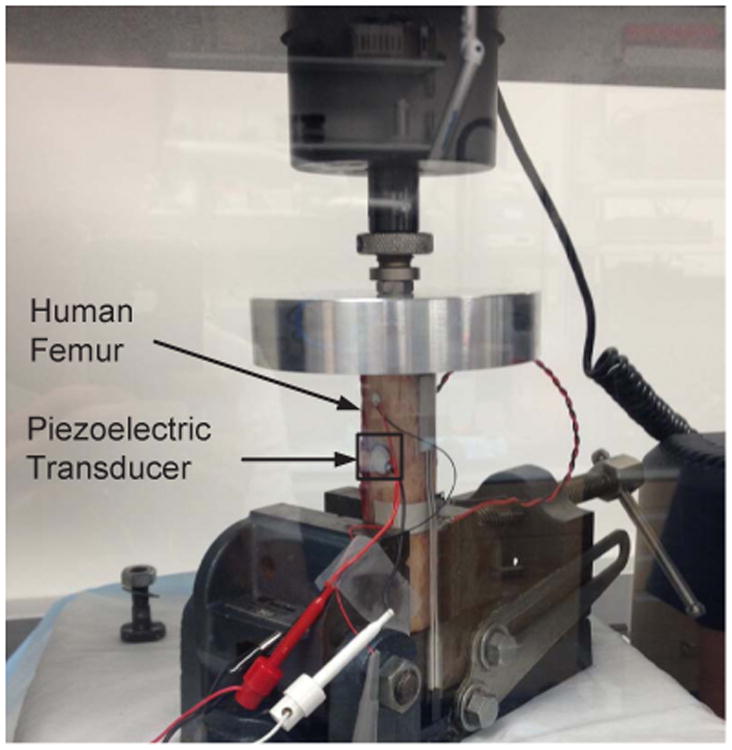
An experimental setup using human cadaveric femur with the proposed injector and a ceramic piezoelectric transducer.

**Fig. 16:**
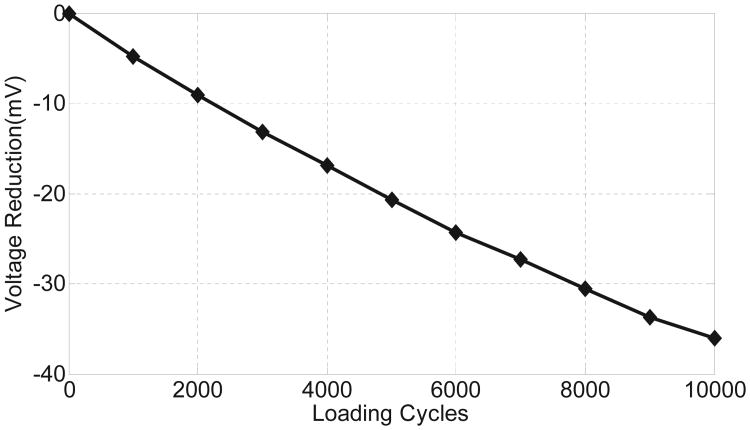
Measured response of the injector when the cadaver setup is subjected to cyclic loading.

In the last set of experiments the fabricated p-HEI sensor and a ceramic piezoelectric transducer (material: PZT-5H) was attached to the surface of a cadaver femur. The experimental setup is shown in Fig. 15. A force of 800 N was applied cyclically to the top of the bone with a frequency of 1 Hz using an Instron 5866 mechanical testing system. The compressive force induces strain-variations in the piezoelectric transducer which then powers the p-HEI sensor. For this experiment, the injector sensitivity was initialized to operate for loading cycles greater than 10000. The measured response is shown in Fig. 16 which also verifies the quasi-linear response of the proposed injector. Note, that the magnitude and the type of loading applied to the bone is much lower than what would be expected under typical service loads. Therefore, the injector should also be responsive under normal operating conditions.

## V. Discussions

**Fig. 17:**
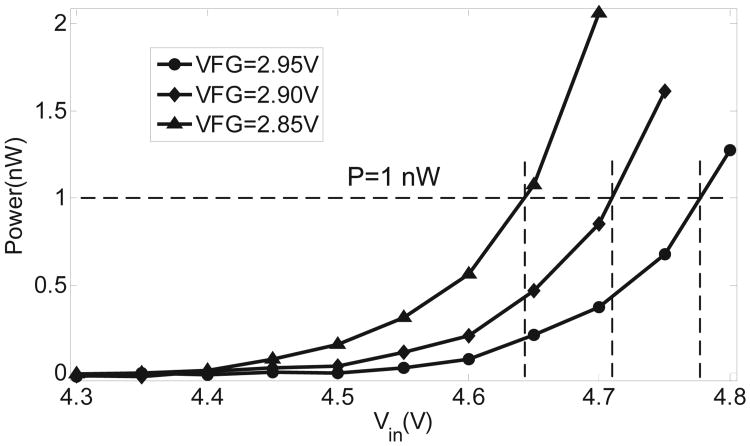
Effect of biasing and diode-chain on the activation threshold of the injector.

In this paper, we presented the design of an ultra-low power CMOS hot-electron injector that can be used for long-term, self-powered monitoring of mechanical strain variations. When compared to the previously reported hot-electron injectors, the proposed injector can operate at 5 nW which is an order of magnitude lower, as is shown in the comparison Table III. This improvement in energy efficiency has been achieved by combining the physics of an energy transducer with the injection response of a pMOS floating-gate transistor when operating in a constant power mode. The quasi-linear response achieved by the injector is important because the circuit could be used for accurate counting and recording of events of interest [10]. On the other hand, the 5 nW operating limit makes the injector suitable for designing structural health monitoring sensors that can be embedded and implanted inside structures [20]. Another attractive feature of the proposed injector is the ability to adjust the minimum power that can activate the injection process. As illustrated by equation 2, the injection efficiency is an exponential function of the source voltage *V_s_*, which implies larger input voltage results in a higher injection efficiency. Therefore, by programming the charge stored on the FG node, we can adjust the injection efficiency and the power threshold. As shown in Fig. 17, for a fixed input power, a larger initial *V_fg_* corresponds to a larger source voltage required for activation.

Note that the 5 nW power limit can be improved further by considering the power dissipation through the diode chain. In this work we have used six diodes in the chain, as a result when the input voltage is 4.5 V, the current drawn through the chain is approximately 0.5 nA. While increasing the number of diodes might reduce this current and hence the power dissipation, it also makes the injector susceptible to damage due to high-voltage electrostatic events. This problem could be mitigated by using diodes with larger sub-threshold slopes and lower leakage currents.

## Figures and Tables

**Table I:** Measured model parameters

Parameter	Value
C	10 nF
*I*_0_	3.5 × 10^−24^A
*κ*	1.1
*U_T_*	0.026 V
*V_inj_*	0.16 V
*β*	1.8 × 10^−21^
*C_T_*	1 pF

**Table II:** Specifications of the prototyped circuit

Parameter	Value
Technology	0.5-*μ*m CMOS
Die Size	1.5 × 1.5 *mm*^2^
Layout Size	200 × 200 *μm*^2^
Minimum Current	< 1 nA
Diode Current	< 0.5 nA
Power Limit	< 5 nW

**Table III:** Comparison of different self-powered mechanical activity monitors

work	power
[9]	800 nW
[11]	540 nW
[19]	200 *μ*W
This work	5 nW
